# Combination of a rotating tube sample divider and dynamic image analysis for continuous on-line determination of granule size distribution

**DOI:** 10.1016/j.ijpx.2019.100029

**Published:** 2019-08-12

**Authors:** Annika Wilms, Klaus Knop, Peter Kleinebudde

**Affiliations:** a Heinrich Heine University Düsseldorf, Institute of Pharmaceutics and Biopharmaceutics, Universitätsstraße 1, 40225 Düsseldorf, Germany; b INVITE GmbH, Drug Delivery Innovation Center (DDIC), Chempark Building W32, 51368 Leverkusen, Germany

**Keywords:** CQA, critical quality attributes, CM, continuous manufacturing, PAT, process analytical technologies, FDA, U.S. Food and Drug Administration, RCDG, roll compaction/dry granulation, TSG, Twin Screw granulation, GSD, granule size distribution, SY, symmetry factor, DIA, dynamic image analysis, Q3, volume-based cumulative undersized curve, Granule size distribution, Representative sampling, Process analytical technologies, Dynamic image analysis, Continuous manufacturing, Process monitoring

## Abstract

The granule size distribution is a critical quality attribute of granules. It has a great impact on further packaging or processing. Due to increasing interest in continuous manufacturing techniques, it is of high interest to develop an in-line or on-line tool to monitor the granule size distribution. However, development of an in-line measurement tool for granule size distribution was challenging since large throughput and inhomogeneous product stream are limiting factors for current particle size analyzers. In this study, continuous sampling was tested in conjunction to a continuous on-line method of size determination using dynamic image analysis. A rotating tube sample divider was used to split previously compacted material in representative samples at different ratios and the sample was directly conveyed to the particle size analyzer where the granule size distribution was determined. The method was tested for different granule sizes to determine limits of detection and its ability to detect these changes immediately, as this enables real-time monitoring of the process. This research is the base for development of control tools concerning the granule size distributions for continuous granulation processes.

## Graphical abstract

**Figure:**
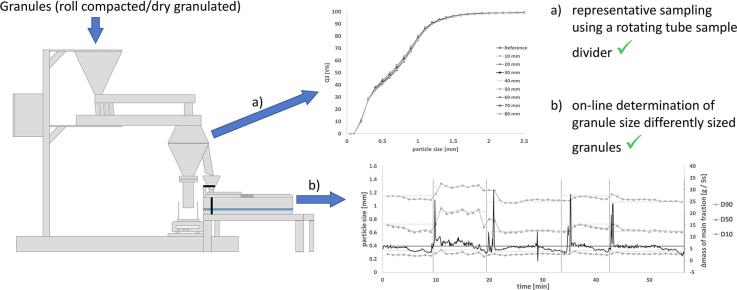

## 1 Introduction

### 1.1 Continuous manufacturing of solid oral dosage forms

Batch processes are defined as processes, in which material is loaded, processed and stored until the next manufacturing step. During storage time, critical quality attributes (CQA) are measured, evaluated and the batch is released for further processing. Disadvantages of batch manufacturing include the necessity to discard the whole batch if a CQA does not match the requirements, that the time to produce a final market form can take several weeks and scale-up may be challenging (Plumb, 2005).

Currently most pharmaceutical products are produced in batch processes, even though numerous benefits of a continuous manufacturing (CM) approach, as commonly seen in food production, are already well known (Plumb, 2005, Rantanen and Khinast, 2015). The interest in CM was furthermore increased by the FDA promoting incorporation of continuous processes in pharmaceutical manufacture (Lee, 2016).

Process analytical technologies (PAT) – tools are aiming to ensure that the CQA's are met during manufacturing (Lee, 2016). If the CQA's do not match the requirements only the material that showed insufficient parameters has to be discarded (Singh et al., 2012). If a deviation from the target values is detected, the process should be adapted to match the target values using control loops. The implementation of PAT is of great importance in order to test the intermediates and ensure quality. The aim is a CM line with sufficient process and product control tools to allow a real-time release of the final drug product.

### 1.2 Granulation

Granulation is one of the most important processes in manufacturing of solid oral dosage forms. With growing interest in CM, granulation processes in which material is fed, processed and discharged continuously are in focus of developmental efforts. Two granulation processes that have gained interest rapidly are roll compaction/dry granulation (RCDG) and twin-screw wet granulation (TSG).

RCDG is a well-established granulation process used in pharmaceutical manufacturing (Kleinebudde, 2004). It has retained its relevance through various innovations due to a number of advantages. These include the ability to process moisture-sensitive active pharmaceutical ingredients and economic benefits through its high energy efficiency (Parikh, 2016, Shlieout et al., 2000). Furthermore, it is exceptionally well-suited for continuous manufacturing (Fonteyne et al., 2015).

Use of an extruder to granulate was published in 1986 (Gamlen and Eardley, 1986). In contrast to other methods of wet granulation, twin-screw granulation (TSG) allows continuous inlet of powder and granulation liquid, processing and discharge (Vercruysse et al., 2012). In contrast to RCDG, drying of the granules is necessary. The dryer can be linked to the outlet of the extruder or incorporated into it (QbCon®, L.B. Bohle, Germany). With growing interest, there is an increasing number of publications on the development of in-line process control tools for TSG (e.g. Harting and Kleinebudde, 2018, Madarász et al., 2018).

Whether granules are packaged or further processed, the granule size distribution (GSD) is a critical quality attribute. The GSD affects the granules flow properties as well as its compaction behavior. Different granule sizes correspond to different particle volumes that can change the fill of tablet press dies and therefore change the resulting tablet strength, disintegration and dissolution. For solely filling processes (e.g. as commonly seen in stick pack or sachet manufacture) a varying particle volume can interfere with volumetric filling and affect the product uniformity of mass and content of single dose preparations (Shekunov et al., 2007). Therefore, it is of great interest to monitor key parameters of the GSD during continuous granulation.

### 1.3 Granule size analysis

There is a variety of analytical tools to measure particle size distributions that can be applied to granules (size ranges e.g. 20–3000 µm (Almeida-Prieto et al., 2004)). There have been multiple efforts to develop monitoring tools of particle size distributions for various pharmaceutical processes. Well-established technologies that have been applied are laser diffraction (Chan et al., 2008) and dynamic image analysis (Madarász et al., 2018, Nalluri et al., 2010). Newer technologies include focused beam reflectance (Greaves et al., 2008) and spatial filter velocimetry (Mangal et al., 2016, Närvänen et al., 2009, Petrak, 2002).

Difficulties occurring specifically in RCDG processes are the high product throughput and the broad, bimodal GSD of the granules (Parikh, 2016). Therefore, direct measurements of the GSD could not be applied to a RCDG process so far. Published efforts to determine the GSD in a RCDG process include correlating the particle size to the slope of in-line obtained NIR spectra (Gupta et al., 2004), off-line use of the Eyecon system (McAuliffe et al., 2015) and spatial filter velocimetry (Mangal et al., 2016).

Dynamic image analysis (DIA) is a valuable tool to determine the GSD of various products. Additionally to the particle size measurement, various particle shape parameters can be monitored. With an appropriate measurement size range, the bimodal GSD curve of dry granulated material can be measured.

### 1.4 Representative sampling

The product stream of a RCDG process can be inhomogeneous (Mangal et al., 2016) and the typical throughput of a roll compaction process is high. The high throughput was found to exceed the capacity of established particle size analyzers (Wilms et al., 2019). Therefore, obtaining a representative sample is necessary in the development of an on-line direct GSD measurement system. This sample should be of adequate size to ensure a representative particle size distribution.

In theory, it is recommended to sample from a powder when it is in motion and to take samples from the complete powder stream (Allen, 2003). Most commonly used is a sample divider in which the sampling vessels rotate underneath a constant stream of product (e.g. PT100, Retsch, Germany). Small quantities of product are thereby split evenly among the mounted number of sampling vessels. Although this system is suitable for lab scale applications, it cannot be applied to a continuous sampling regime. An opposite approach, a rotating product stream and a static sample exit is also commercially available (e.g. PT35-K, Vock Maschinen- und Stahlbau GmbH, Germany) but has not been used in pharmaceutical applications yet.

Another commercially available approach for in-line sampling is the “TWISTER” system (Sympatec GmbH, Germany). In this system, a sampling tip facing the opposite direction of the product stream is placed in the product stream. Therefore, product is collected if it falls directly on the sampling tip. To ensure the whole cross section of the product stream is sampled the sample tip is driven along defined trajectories around the product stream tube (Witt et al., 2004). However, there are no publications confirming the representative sampling of pharmaceutical bulk products in peer-reviewed journals so far.

### 1.5 Objectives

The aim of this study was to implement an in-line rotating tube sample divider to representatively split granules that were produced using RCDG and to determine the particle size of the sample on-line in real-time. It should be shown, whether the sample splitter can split representatively at various sampling ratios. The material throughput should be at least 5 kg/h in total to ensure relevance for pharmaceutical production. The focus was to obtain reliable data of the GSD and to investigate, whether the system is sensitive to changes in the material granule size. The tools settings were optimized for use in continuous manufacturing of pharmaceutical products. The method should then be universally applicable to continuous granulation processes.

## 2 Materials and methods

### 2.1 Materials

**Fig. 1:**
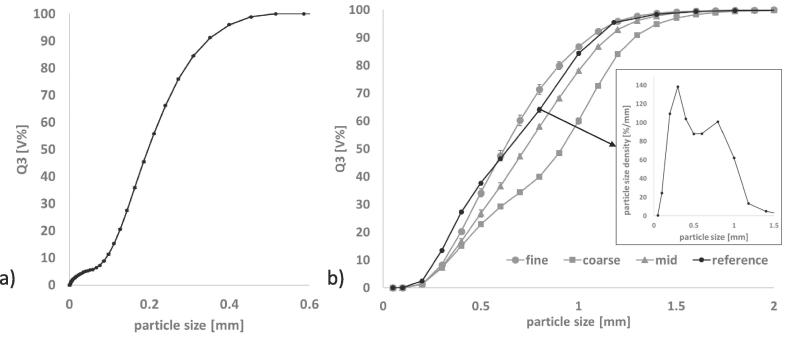
Cumulative size distribution a) DCPA excipient b) DCPA granules produced using BRC 25. Measured with Haver CPA 2-1. In box: particle size density distribution of reference granules. n = 3; mean ± sd.

Dibasic calciumphosphate anhydrous (=DCPA) (DiCaFos® A150, Budenheim, Germany) was used for all experiments. The particle size distribution of the excipient measured with laser diffraction (Mastersizer 3000, Malvern Panalytical, United Kingdom) is shown in Fig. 1a).

### 2.2 Roll compaction/dry granulation

DCPA was used for RCDG on a BRC 25 (LB Bohle GmbH, Germany). The roll compactor was equipped with smooth surfaced rolls, rim roll sealing system and a 360° rotating turbo sieve (BTS, LB Bohle GmbH, Germany). The specific compaction force was varied to obtain granules of different granule size distributions (Fig. 1b)). Granules were produced at specific compaction forces between 5 kN/cm and 18 kN/cm. As a correlation between the specific compaction force and respective granule size is of no importance for this work, the granules are hereafter referred to as fine grade, coarse grade, mid grade (Fig. 1b)). Reference granules were used for initial set-up of the equipment, confirming the representative sampling and evaluating measurement settings.

### 2.3 In-line representative sampling of granules produced using RCDG

**Fig. 2:**
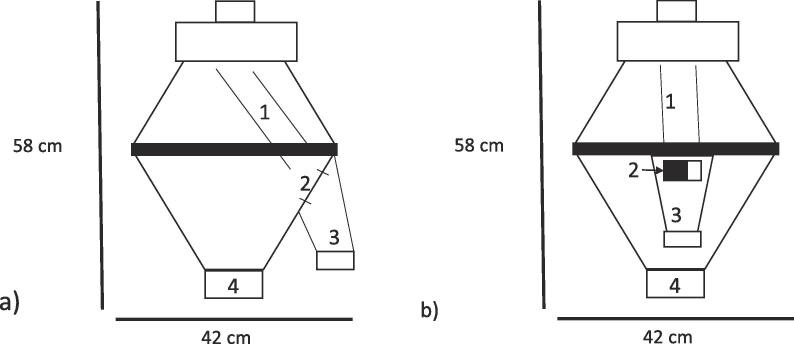
Rotating tube sample divider PT35-K. 1 – divider tube, 2 – adjustable gap, 3 – sample outlet, 4 – main product outlet a) gap at the side b) gap at the front (black = closed; white = open).

PT35-K (Vock Maschinen- und Stahlbau GmbH, Germany), a rotating tube sample divider, was used for all experiments. The divider tube rotates at a speed of 29 rpm. It moves closely along the wall of the lower cone. An adjustable gap that can be closed or opened up to 80 mm is located inside the lower cone (Fig. 2).

**Fig. 3:**
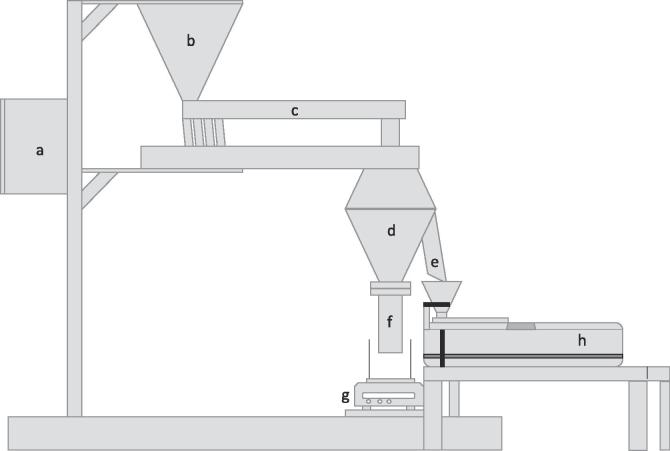
Setup of PT35-K for off-line measurements. a) control unit b) input funnel c) vibration chute d) biconical sample splitter e) sample outlet f) main fraction outlet g) balance h) Haver CPA 2-1.

The PT35-K was instrumented with a control unit (Fig. 3).

It had to be confirmed whether the PT35-K splits representative samples from a bulk of granules that was produced using RCDG. The adjustable gap includes a scale of 10 mm steps. Therefore, to ensure consistent sampling gap widths, experiments were conducted increasing the gap width 10 mm for each setting (0–80 mm). A bulk of reference granules (approximately 2 kg) was split and the GSD of the sample was determined off-line using Haver CPA 2-1 at an optical density range of 0.8–3. The GSD was determined in triplets and the sample was reunited with the main fraction in order to reobtain the complete bulk. To determine the GSD of the total bulk it was split using an established rotary sample divider (PT 100, Retsch, Germany). The GSD of the bulk was determined at three points during the experiment to evaluate whether mechanical stress causes breakage of the granules during the experiment.

### 2.4 Granule size determination

The Haver CPA 2-1 (Haver & Boecker, Germany) determines granule size based on a CCD line scan camera that scans particles in backlight of a LED light source. The line scan camera records with a frequency of up to 50 million pixel-scans per second. The scanned lines will then be joined to a dataset (picture) and evaluated in real-time. Further technical information is presented in Table 1. Particles are transported and separated using a vibration chute and, if needed, additional ultrasound. The separated particles pass through the sight of the line camera in free fall. This analysis is a non-destructive measurement method and the measured sample can be reunited with the main fraction afterwards.

**Table 1:** Haver CPA 2-1 technical information.

Feature	Haver CPA 2-1
Measurement range	34 µm–25 mm
Sensor	CCD line scan camera
Pixel count	2048
Feeder width	65 mm
Pixel frequency	50 MHz
Light source	LED, red
L × W × H	800 mm × 200 mm × 355 mm
Environment temperature	5–40 °C
Maximum surrounding humidity	85%

Granule size calculation was based on the equivalent diameter. Each pixel has a height and width of 34 µm. For every experiment and product, the optical density range was evaluated. The optical density range chosen was 0.8–3.0. The optimal optical density range of 0.8–3.0 originates from recommendations from the analyser manufacturer for usage of the system in lab scale experiments. A low optical density range will result in a slower feed rate and less particles will be delivered to the measurement area. A higher optical density however allows to deliver more particles to the measurement zone in the same time interval. After measurements in triplets the optical density range was increased step-wise until significant differences in GSD were recorded. During optimizing of the measurement settings, the mass of the sample and the measurement time was noted. This allowed calculation of the measurement speed.

Haver CPA 2-1 analyses the shape of each individual particle and calculates a symmetry value (SY). It is calculated using the smallest ratio of all symmetry axes that run through the gravimetrical centre of the particle projection area. Register a SY value of one (“1”) is a characteristic of a perfect sphere and will also for a particle that has the size of one pixel. Therefore, the SY value is also dependent on the resolution. If two particles are not separated sufficiently, there is a possibility of them touching during their free fall through the sight of the camera. Hence, they will be treated as one particle with a bigger size for analysis. If the common area, in which two particles overlap is small, the recognized particle will show a low symmetry factor. In the following experiments, results were recorded using no SY restrictions or filters. Afterwards, to evaluate the impact of introducing a minimal SY requirement (filter), the Haver CPA raw-data was recalculated to include only particles with a SY of 0.5 or more.

### 2.5 On-line analytics

The input funnel of the Haver CPA 2-1 was positioned underneath the sample outlet of the PT35-K (Fig. 3) for on-line experiments. The granules were poured in the PT35-K input funnel, split in the PT35-K and subsequently measured using Haver CPA 2-1. All settings were chosen prior to starting the measurement and could not be adjusted during the measurement. Temporary measurements were conducted for one minute each. A lab scale balance (CPA5201, Sartorius AG, Germany) was placed underneath the outlet of the main fraction. The mass is tracked (Sartorius connect, Sartorius AG, Germany) in 5 s intervals.

### 2.6 Statistical analysis

All off-line measurements were conducted at least threefold. To determine, whether different samples show the same GSD, their D10, D50 and D90 values were compared. An F-test was executed to compare the variances. Depending on the outcome of the F-test a two-sided *t*-test (α = 0.05) was conducted for either equal or unequal variances.

## 3 Results and discussion

### 3.1 In-line representative sampling of granules produced using RCDG

**Fig. 4:**
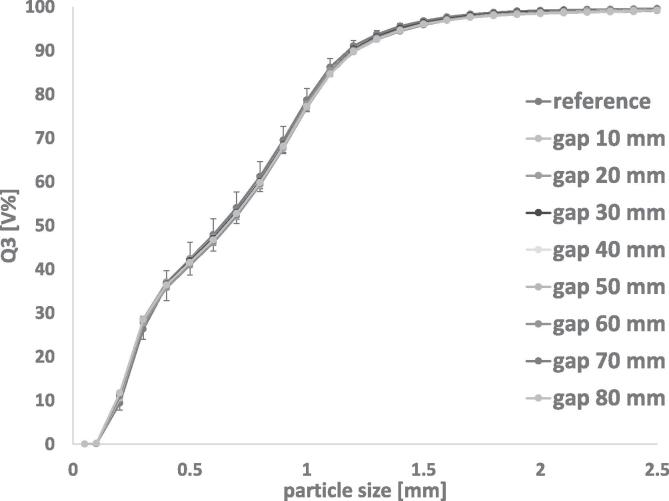
Cumulative size distributions of DCPA granules. Optical density = 0.8 – 3.0; n = 3; mean ± sd. reference: n = 3 × 3; mean ± sd.

It had to be verified whether PT35-K splits representative samples from a bulk of RCDG granules. The GSD of the reference and the samples, depending on gap width and thereby the split ratio, is displayed in Fig. 4. Furthermore, the split ratios were determined and compared to the values provided by the manufacturer (Table 2).

**Table 2:** Split ratios PT35-K.

gap opening [mm]	split ratio (stated in handbook)	split ratio (measured)
10	1:80	1:66
20	N/A	1:37
30	1:41	1:27
40	1:28	1:21
50	1:16.5	1:17
60	1:14	1:14
70	1:12	1:12
80	1:10	1:11

It was observed that the split ratios differ from the values stated in the PT35-K handbook. For further experiments, the determined values were used.

The reference was measured in triplets at three time points during the experiment. The resulting mean GSD curve did not differ significantly to the GSD curves of the samples. However, the reference showed larger standard deviations (Fig. 4). A decrease in particle size during the experiment was observed. This was expected, as the same granules were stressed repeatedly in conveying and rotating tube sample dividing as well as conventional sample splitting. Regarding the amount of stress on the granules, the decline in granule size is negligible. For regular application, a single mechanical stress is not regarded as problematic. This proved representative sampling for granules obtained using RCDG at different gap widths and split ratios between 1:66 and 1:11 (Table 2).

### 3.2 Off-line determination of measurement settings

**Fig. 5:**
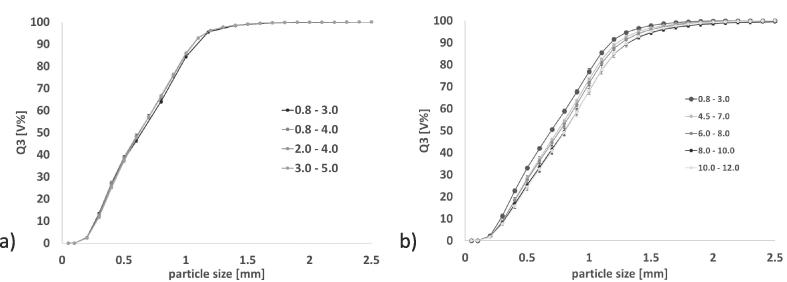
Cumulative size distributions of reference granules. Varying optical density. No symmetry restriction. a) Experiment A b) Experiment B. n = 3; mean ± sd.

The critical measurement setting of optical density range was defined in the Haver CpaServ software prior to starting the measurement. No adjustments to the predefined range could be made after the measurement had started. Therefore, it was critical to define this range carefully. Two major factors were considered. The first factor was the significant shift in GSD parameters that could be observed when the optical density range was increased (Fig. 5). Secondly, a high throughput of material, which correlates to an increased optical density and a high measurement speed, was desirable in order to ensure relevance for pharmaceutical production. The aim was to measure as much material as possible without results deviating significantly from the reference recorded at an optical density of 0.8–3.0. The settings for on-line experiments were determined using the reference granules shown in Fig. 1b).

#### 3.2.1 Deviation from reference measurement at increasing optical densities

GSD curves obtained using varying optical density ranges are shown in Fig. 5. The granules measured in Fig. 5a) were the reference granules for in-line experiments (Fig. 1b)). Optical density could be increased from 0.8–3.0 to 3.0–5.0 without significant differences (experiment A). In a second experiment (experiment B) the optical density was increased until a significant deviation from the reference was observed (Fig. 5b)). Unfortunately, before these measurements were conducted the granules were also used for further preliminary testing and, as a result from repeated mechanical stress, the particle size had decreased. The results are however indicating that starting from an optical density of 4.5 – 7.0 the GSD curve shows a significant shift compared to the reference measurement. As expected, the granule size is overestimated at high optical densities due to more particle overlapping and agglomerates in the measurement channel.

#### 3.2.2 Determination of measurement duration

Samples were measured at different optical densities and the measurement time was recorded to determine the measurement speed (Table 3). Each sample was measured threefold for each setting. Knowledge of the measurement speed was critical as the system parameters are evaluated for this throughput of material. The complete product conveyance and the sampling ratio should then provide a sample input into Haver CPA 2-1 equal to the previously determined measurement speed.

**Table 3:** Speed of measurement. No asterisk marks results from experiment A, the asterisk indicates results from experiment B. n = 3; mean ± sd.

Optical density	Sample mass [g]	Time of measurement [s]	Speed of measurement [g/min]	Number of particles [*10^6^]
0.8–3.0*	37.7	536 ± 35	4.22 ± 0.28	1.53 ± 0.06
0.8–3.0	76.3	1034 ± 138	4.43 ± 0.60	3.10 ± 0.04
0.8–4.0	76.3	829 ± 96	5.52 ± 0.65	3.18 ± 0.05
2.0–4.0	76.3	661 ± 5	6.93 ± 0.05	3.10 ± 0.00
3.0–5.0	76.3	486 ± 8	9.42 ± 0.15	3.06 ± 0.04
4.5–7*	36.8	154 ± 3	14.37 ± 0.28	1.25 ± 0.01
6.0–8.0*	36.7	135 ± 9	16.27 ± 1.08	1.07 ± 0.05
8.0–10.0*	35.5	100 ± 2	21.30 ± 0.96	0.78 ± 0.09
10.0–12.0*	35.6	82 ± 1	26.05 ± 0.19	0.67 ± 0.1

**Fig. 6:**
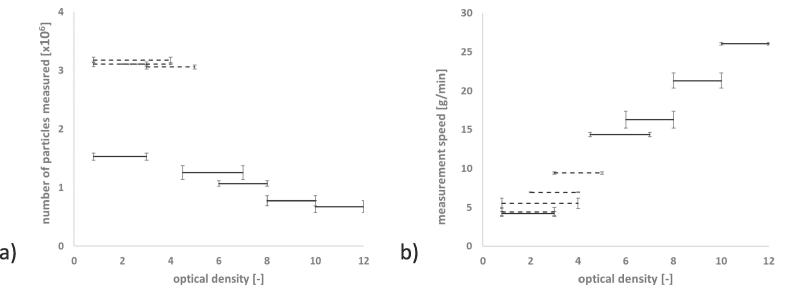
a) Total number of analysed particles and b) measurement speed plotted against the bottom (black) and top (grey) optical density range, experiment A (dashed line), experiment B (solid line) n = 3; mean ± sd.

In Fig. 6a), the total number of particles, that were analysed are plotted against the optical density setting chosen. Although the number of particles in the sample was not changed, the number of particles measured varied significantly. Less particles were registered at increasing optical density ranges. A high optical density required more particles in each picture taken by the analyser. To achieve this, the increased mass flow also increased chances of two or more particles touching, overlapping or agglomerating. They were then analysed as one particle and the total number of particles decreased. Experiments A and B were conducted with samples of different masses and hence differ in total amount of particles.

The measurement speed plotted against the optical density is shown in Fig. 6b). As expected, an increase in measuring speed could be observed with increasing optical density ranges. It is important to note, that two experiments using granules of different granule sizes and sample masses led to similar results and trends. In the future, this should be confirmed for different materials and granule sizes. This could then allow prediction of the measurement speed depending on optical density settings and material properties.

#### 3.2.3 Symmetry settings

**Fig. 7:**
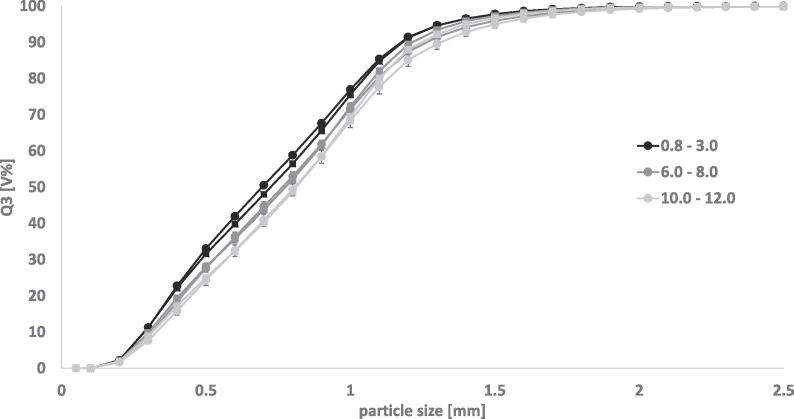
Cumulative size distributions of reference DCPA granules, n = 3; mean ± sd. Varying optical density. Symmetry restriction: circle = no restriction, square = minimum SY of 0.5.

The influence of varying the symmetry setting was evaluated (Fig. 7). As expected, the GSD curve was shifted to larger granule sizes with increasing optical density ranges. Introducing a minimal symmetry factor of 0.5 adjusted the GSD curves to smaller sizes, however, the effect was not large enough to correct the curves to the reference measurement. After addition of symmetry limitation, the curves still varied significantly from their reference. For all further experiments, no symmetry restrictions were applied.

Using this data, the expected throughput for the main fraction was calculated. Taking the data of the optical density setting 2.0–4.0 (Table 3) into account, a measurement speed of 7.2 g/min was determined. For a gap width of 80 mm the sampling ratio was determined to be 1/11 (Table 2). Throughput was calculated to be 79.2 g/min (≈4.75 kg/h) and is in relevant production throughputs of commercial roll compactors. However, the mass that was tracked during the experiments was solely the mass of the main fraction (72.0 g/min). As the mass was tracked every 5 s, the desired mass signal resulted in 6.3 g/5s.

### 3.3 On-line determination of GSD using DIA

**Fig. 8:**
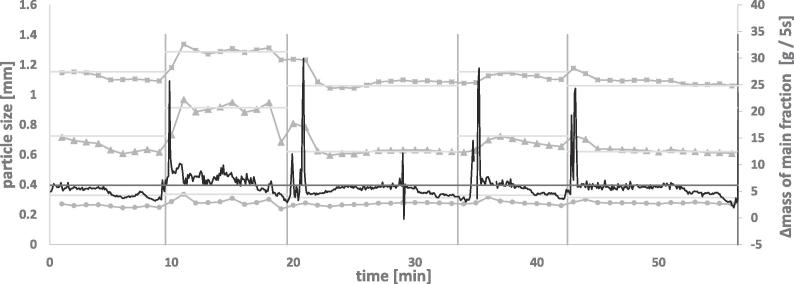
Measured GSD quantiles. squares = D90, triangles = D50, circles = D10. Roman numbers indicate the sample that was measured at the given time period. I = mid, II = coarse, III = fine. Measurement duration = 1 min. Dark black = feed rate.

Fig. 8 shows results of an on-line measurement (off-line results of the used granules can be seen in Fig. 1b)). The fine and the coarse grade of granules were separated representatively into two batches. When all material was transferred on the vibrating chute, no more material was present in the input funnel, the granules were brushed forward. This was done in order to avoid a decline in throughput based on low fill-level of the chute and mixing of the different grades when the new grade was poured in the funnel. However, both factors could not be eliminated completely. Hence, they were expected to influence the measurements and lead to disturbances after changing the granule type. Brushing forward the material also had an impact on the feed rate and should be avoided in future experimental set-ups.

The vertical lines in Fig. 8 indicate the time points at which the new grade of granules was filled into the input funnel. The horizontal lines indicate the off-line determined reference values for D90, D50 and D10.

Based on the GSD measurements, it was possible to distinguish the differently sized granules. The time needed until changes in the GSD quantiles could be recorded was less than two minutes. This is understandable as the granules did not reach the sample splitter directly but were conveyed in the vibration chute of the PT35-K instrumentation and in the vibration chute of Haver CPA 2-1 first. Residence time in the splitter was negligible. Therefore, the differences in GSD could be observed close to real-time.

Compared to off-line measurements, the D10 value was underestimated for most measurements. This could be explained based on the settings that were chosen. As big granules and fines require different settings (namely optical density) the chosen settings were a compromise in which all key quantiles can be measured in realistic scales. A lower optical density range, as the fine fraction would require for measurement, would also require a lower material throughput. The material throughput however, is crucial for ensuring this measurement system is relevant for pharmaceutical production. As the D50 is, arguably, the most important parameter it was important to measure the D50 precisely. The slight underestimation of the D10 was assessed as less critical.

Fluctuations were detected during the measurements of the GSD quantiles. Plotting of the mass flow (secondary y-axis) against the time shows the mass flow throughout the whole experiment (Fig. 8). It can be seen that the mass flow fluctuated frequently, especially in the beginning and at the end of a new bulk that was conveyed. Comparing these trends, e.g. a decrease in mass flow at minutes 6–10 lead to fluctuations in the GSD parameters. As previously discussed, the expected feed rate based on the main fraction mass was 6.3 g/5s (black horizontal line, Fig. 8). If this throughput was reached, the measurements correlated well with the reference values (see minute 25–30). The settings were optimized for a certain throughput (7–9 g/min sample and 72–92.4 g/min bulk). A deviation from this throughput changed the requirements for the optical settings and lead to different results. An option to adjust the settings with a varying throughput would be beneficial for these measurements.

## 4 Conclusions

Representative sampling of granules produced by RCDG was successfully implemented using a rotating tube sample divider. This enables on-line characterizations that could not be conducted previously due to limited operational capacity of the measurement or due to the inhomogeneity of the RCDG-product stream. The sampling procedure can be integrated in a bypass solution for an on-line measurement.

The sampling was joined to a particle size analyzer based on dynamic image analysis, which measured every granules size and shape. In future, monitoring of the granule size can be enhanced further by development of control tools. Real-time adjustments to the measurement settings according to the current product mass flow could enhance this set-up.

RCDG was simulated to produce granules at a throughput of 5 kg/h and with sampling an amount of 10%. As the sample divider allows for smaller splitting ratios even higher throughputs can be aimed at using this system.

The experimental system can be transferred to a RCDG process and validated.

## Funding

This research did not receive any specific grant from funding agencies in the public, commercial or not-for-profit sectors. This work was supported by the Drug Delivery Innovation Center (DDIC), INVITE GmbH, Leverkusen.

## Declaration of Competing Interest

The authors declare that they have no known competing financial interests or personal relationships that could have appeared to influence the work reported in this paper.
